# International socioeconomic inequality drives trade patterns in the global wildlife market

**DOI:** 10.1126/sciadv.abf7679

**Published:** 2021-05-05

**Authors:** Jia Huan Liew, Zi Yi Kho, Rayson Bock Hing Lim, Caroline Dingle, Timothy Carlton Bonebrake, Yik Hei Sung, David Dudgeon

**Affiliations:** 1 Division for Ecology and Biodiversity, School of Biological Sciences, The University of Hong Kong, Pokfulam, Hong Kong SAR.; 2 Science Unit, Lingnan University, Tuen Mun, Hong Kong SAR.; 3 School of Life Sciences, The Chinese University of Hong Kong, Sha Tin, Hong Kong SAR.; 4 Department of Biological Sciences, National University of Singapore, 16 Science Drive 4, Singapore 117558, Republic of Singapore.

## Abstract

The wildlife trade is a major cause of species loss and a pathway for disease transmission. Socioeconomic drivers of the wildlife trade are influential at the local scale yet rarely accounted for in multinational agreements aimed at curtailing international trade in threatened species. In recent decades (1998–2018), approximately 421,000,000 threatened (i.e., CITES-listed) wild animals were traded between 226 nations/territories. The global trade network was more highly connected under conditions of greater international wealth inequality, when rich importers may have a larger economic advantage over poorer exporting nations/territories. Bilateral trade was driven primarily by socioeconomic factors at the supply end, with wealthier exporters likely to supply more animals to the global market. Our findings suggest that international policies for reducing the global wildlife trade should address inequalities between signatory states, possibly using incentive/compensation-driven programs modeled after other transnational environmental initiatives (e.g., REDD+).

## Teaser

Wealth inequality between nations influences patterns in the international wildlife trade.

## INTRODUCTION

The wildlife trade is one of the main causes of species loss (*1*) in the midst of a sixth mass extinction (*2*). Wild animals are widely commodified (*3*) and subjected to unsustainable harvesting practices (*4*, *5*), leading to precipitous global population declines (*6*). Although the trade mainly affects source ecosystems, it can cause indirect harm to recipient habitats by acting as a conduit for potentially invasive species (*7*, *8*). Moreover, the wildlife trade is a pathway for diseases (*9*) that are capable of damaging vulnerable animal populations (*10*). The amphibian chytrid fungus, *Batrachochytrium dendrobatidis*, for instance, threatens an estimated 200 species of frogs globally (*11*). Pathogenic organisms spread via trade do not only infect animals but may sometimes be transmittable to humans (*12*, *13*). These outbreaks can vary in scale, from localized infection clusters (e.g., the Middle East respiratory syndrome and Ebola) to global pandemics, as recently evidenced by the coronavirus disease 2019 (COVID-19) virus (*13*).

The impacts of the wildlife trade on global biodiversity and human health have understandably motivated collaborative efforts to curtail it. The Convention on International Trade in Endangered Species of Wild Fauna and Flora (CITES) represents one of the most important multinational attempts to regulate the cross-border flow of threatened wildlife. While CITES plays a major role in conserving threatened species (*14*, *15*), its efficacy depends (among other things) on the willingness and ability of signatory states to enact trade controls (*16*). This dependence on adherence and enforcement means that the protection afforded to threatened species may be inadequate in some parts of the world (*16*).

At the local (e.g., national and subnational) level, community engagement initiatives (*17*) are used to compensate for limitations in wildlife trade regulations (*16*, *18*). These strategies often seek to address socioeconomic factors underlying market trends (*19*, *20*), and the approach has been relatively successful (*17*). Similar policies are currently lacking at the global level, however, as multinational wildlife trade frameworks tend toward regulatory efforts (e.g., CITES).

We assessed the role of socioeconomic factors in driving the global wildlife market, with the goal of informing alternative transnational policies for reducing the international trade in threatened species. We focused on trade in CITES-listed, wild-caught animals from 12 widely commodified animal groups over a 21-year period (1998–2018 inclusive), namely, amphibians, anthozoans (anemones, soft and hard corals), arachnids (spiders and scorpions), birds, bivalves, fishes, hydrozoans (e.g., jellyfish and fire corals), insects, mammals, reptiles, sharks/rays, and snails. We collated data from the CITES trade database and standardized trade volumes in terms of approximate number of individual animals before analyses at two scales—global and bilateral (i.e., between exporter-importer pairs). At the global scale, we constructed trade networks for each year in the time period examined and summarized their topology with commonly used indices (e.g., link density and connectance). We then (i) compared trade network topology against contemporary and lagged (i.e., from the previous year) measures of global wealth and wealth inequality. At the bilateral scale, we (ii) identified key socioeconomic drivers of trade volume between exporter-importer pairs among various indicators of national economy, demography, commodity trade, and governance. We repeated our query separately for each of the 12 animal groups analyzed to (iii) investigate differences in trade patterns. Lastly, we (iv) contextualized our findings against the wider international wildlife market, which includes trade in non–CITES-listed species and illegally traded animals, using a subset of our data (i.e., all trade entering the United States between 2000 and 2014).

## RESULTS AND DISCUSSION

### Overall trends

**Fig. 1:**
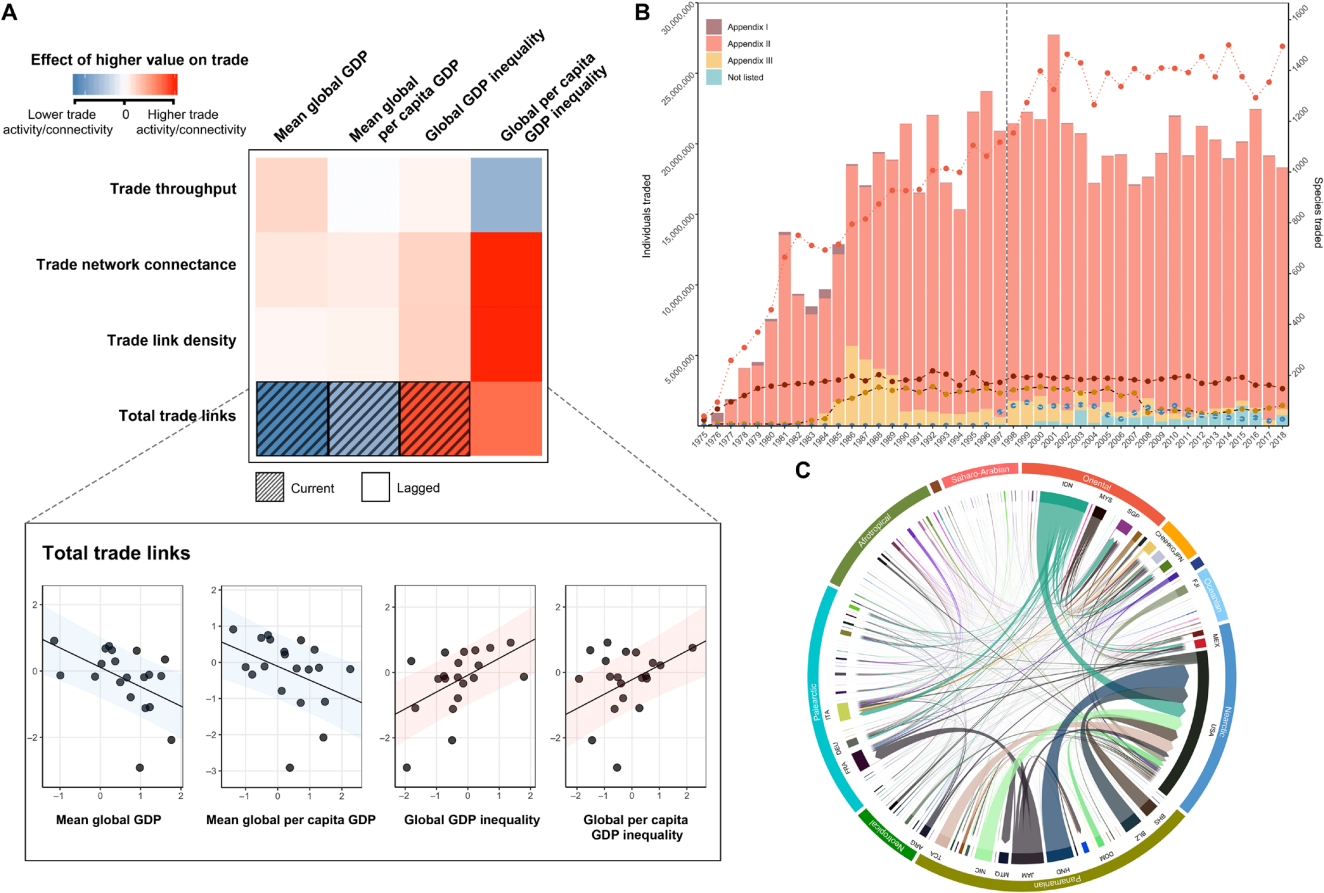
Trends in the international trade of threatened wild animals. A total of 226 nations/territories making up 8342 unique exporter-importer pairs participated in the global market between 1998 and 2018, where trade network connectivity generally increased with international wealth inequality. (**A**) Each tile represents the linear relationship (depicted in inset) between a network index (row) and a measure of global wealth/wealth inequality (column). Tile colors represent slope coefficients indicating the direction and strength of respective statistical relationships, while panel textures identify whether current or lagged (from the previous year) measures of wealth/wealth inequality are more parsimonious predictors of the topology of the global trade network. Shaded regions in line plots within the inset represent 95% credible intervals. (**B**) Dashed line marks the beginning of the time period analyzed in this paper. The number of individuals traded is represented by bars, while dots represent species counts. Appendix numbers in the legend refer to CITES appendices. (**C**) Width of arrows indicates volume of trade flows in terms of estimated number of individual animals. The inner track of the chord diagram represents nations/territories identified by ISO 3166 country codes, while the outer track represents biogeographic region (*21*).

**Fig. 2:**
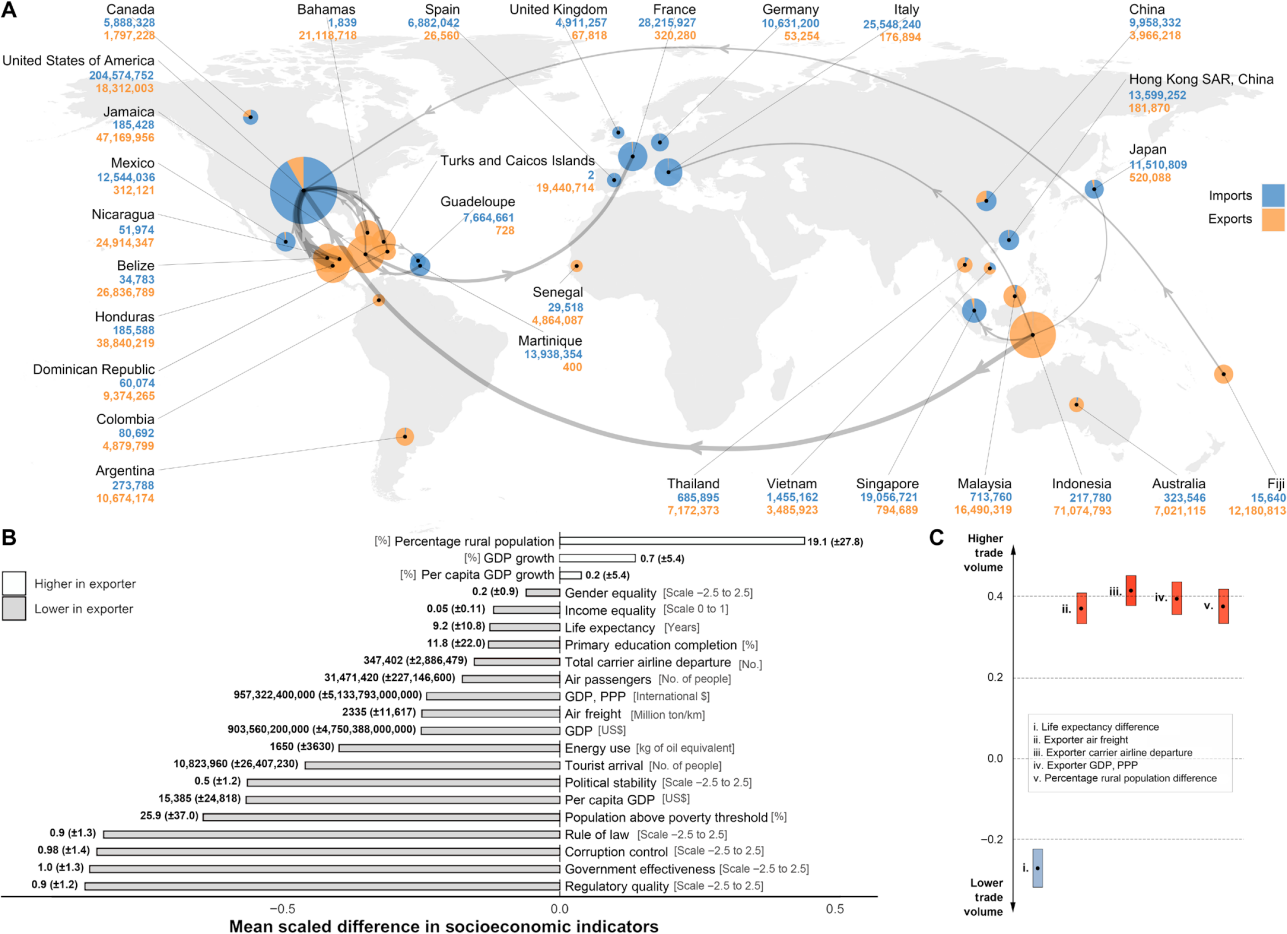
Socioeconomic dimensions of the global wildlife market. Wild animals in the trade mostly flowed from poor developing economies to rich developed nations/territories, but wealthier exporters are sources of higher trade volumes. (**A**) Map of the 30 top participants and the 15 largest trade links in the global wild animal trade between 1998 and 2018. The size of the pie charts and width of the arrows are indicative of total individual animals traded, detailed by numbers associated with each participating nation/territory. (**B**) Bars represent the scaled average difference in socioeconomic indicators between exporters and importers. Actual numerical differences are denoted by the numbers associated with each bar (±SD). (**C**) Data points represent the scaled posterior distribution of slope coefficients (median ± maximum/minimum value within the 95% credible interval) describing the effects of the top five socioeconomic predictors of bilateral trade volume. The slope coefficients indicate the direction and strength of respective statistical relationships.

An estimated 421,696,531 individual wild-caught (CITES-listed) animals were traded between 1998 and 2018 (Fig. 1). The largest exporters of wild animals in the trade between 1998 and 2018 were Indonesia, Jamaica, and Honduras, while the United States was the biggest importer, with France and Italy a distant second and third, respectively (Figs. 1 and 2). At the biogeographical scale (*21*), the Panamanian and Oriental regions were the main sources of wild-caught animals (Fig. 1).

Overall, we found that the wild animal trade network was more highly connected (i.e., higher connectance, link density, and total links) when wealth inequality (overall and per capita) between participating nations/territories was greater (Fig. 1 and table S3). There were also fewer links in the global trade network when average national and per capita wealth were higher (Fig. 1). Conversely, total trade activity (i.e., throughput) was not correlated with measures of global wealth and wealth inequality (table S3). Our data show a lag in the relationship between the global economy and most measures of trade network patterns (Fig. 1).

As the wild animal trade generally flowed from poorer developing nations to rich developed ones (Fig. 2), observed (positive) correlations between wealth inequality and trade connectivity indicate that the global trade network is more extensive (i.e., more connected) when importers have a greater economic advantage over a wider range of exporters. This may be because the wealth disparity between exporting and importing economies makes it more lucrative to sell wildlife products in export markets than it is to trade locally. To take an analogous example, high-grade seafood is commonly exported from developing nations, even if there is local demand for these goods, because rich importers can afford to pay a price premium (*22*). Higher levels of inequality may exacerbate this effect, thus favoring the establishment of a greater number of trade partnerships.

Although exporters were generally poorer than importers, comparatively wealthy nations/territories at the supply end of the trade [i.e., higher gross domestic product (GDP)/purchasing power parity (PPP) and smaller difference in life expectancy relative to importing partner] supplied more animals to the global market (Fig. 2). Moreover, we also found that export volumes scaled positively with air traffic in source nations/territories (Fig. 2). Both observations suggest that bilateral trade volume is driven by export costs, so wealthier source nations/territories with better logistics performance and/or international connectivity are likely to export more animals because the overhead they incur is lower (*23*). Our findings also reflect the increasing ascendency of commercial/industrial operations over subsistence/artisanal practices in the global wildlife trade (*24*), which parallels the dominance of wealthy nations in global fisheries (*25*).

Trade volume appeared to depend on access to natural habitats and wild animal populations (*3*), as more animals were traded between nations/territories with greater disparity in their respective proportion of rural populations. This is likely driven by the combined effect of greater supply from predominantly rural exporters and higher demand from mainly urban importers (Fig. 1B) (*19*).

Socioeconomic indicators that are most predictive of bilateral trade volumes (i.e., the most informative variables determined by Random Forest algorithms) were largely associated with the supply/exporter end of the market, indicating that the system may be supply constrained because international demand for wild animal products currently exceeds export capacities. Our assumption is consistent with the relatively steady volume of wild animals traded over the years despite the increasing supply of captive-bred alternatives (*26*). For example, the persistent demand for wild-caught animals is particularly evident within the pet trade (*27*) and in the traditional Chinese medicine market, where wild-sourced ingredients are perceived as having a higher potency (*28*).

### Differences in trade patterns between animal groups

Unlike in the overall trade (Fig. 1), we observed clear temporal patterns (between 1998 and 2018) in several animal groups. For instance, the number of amphibians, arachnids, birds, fishes, insects, and mammals in the trade declined in recent years (figs. S2, S4, S5, S9, and S10), while the trade in sharks and rays grew rapidly between 2013 and 2018 (fig. S12). The underlying causes of these temporal trends are not always obvious, but steep declines in trade volume of birds after 2005 coincided with the European Union (EU)’s ban on wild-caught individuals aimed at reducing the spread of bird flu (Commission Decision 2005/760/EC). Until 2005, Italy, Spain, and Portugal had been among the largest importers of wild birds (fig. S5) (*29*). For other animal groups, declining trade volumes could be the result of effective enforcement and decreasing demand for products made from endangered animals (*30*, *31*)—or more worryingly, as a consequence of diminishing wild populations, an increasingly dominant illegal trade and widespread “laundering” of illegally traded animals (*32*, *33*). Trade in sharks and rays, in contrast, has increased rapidly since 2013, but this may reflect improved documentation of exports/imports after nine shark species were added to CITES Appendix II between 2014 and 2017 (*34*).

**Fig. 3:**
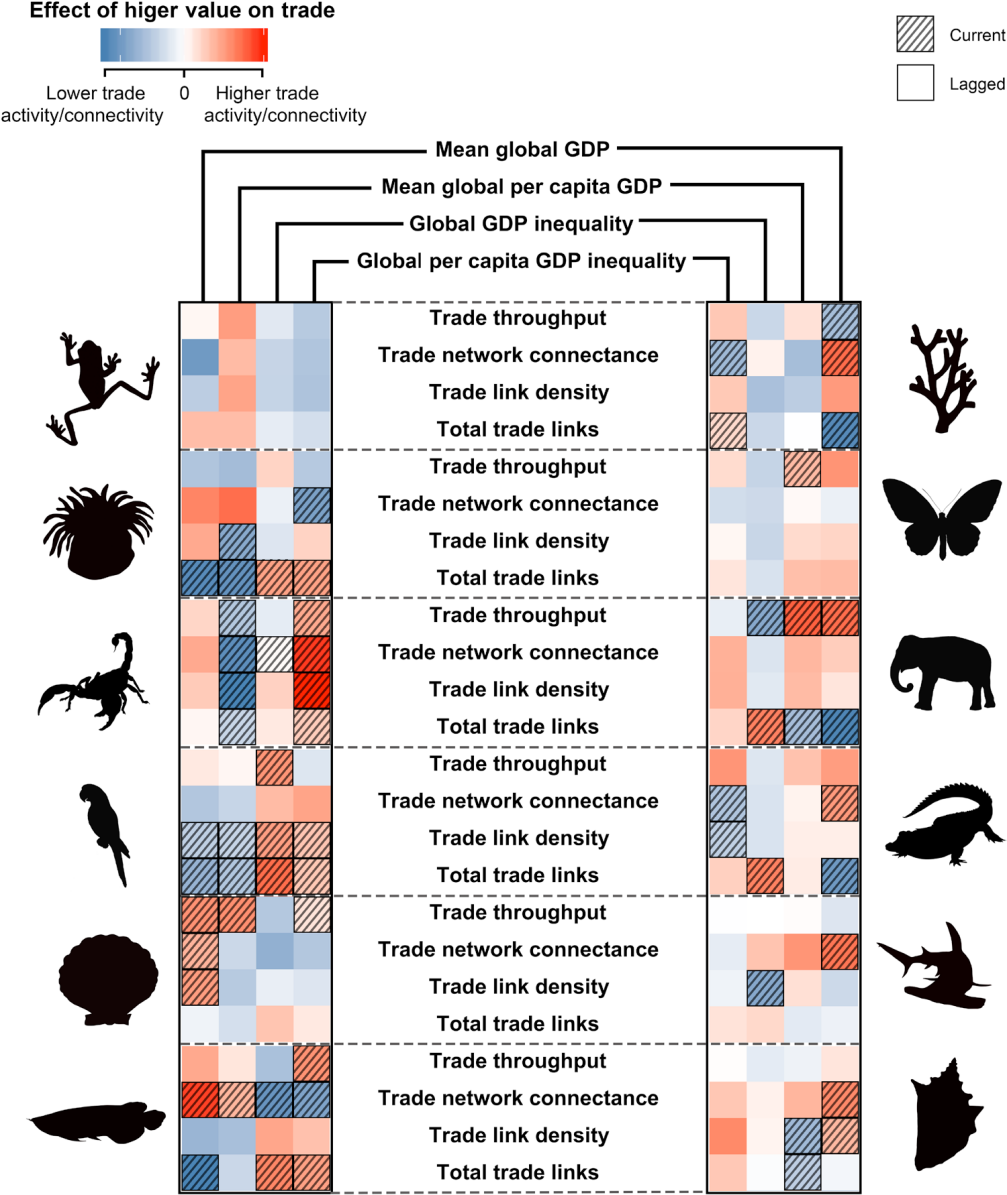
Animal group-specific socioeconomic drivers of the global wildlife trade network. Statistical relationships between group-specific trade networks and indicators of global wealth/wealth inequality were highly variable, but overall trends (Fig. 1) were mirrored in several animal groups. The significance of tile colors and textures is as described in Fig. 1. Groups analyzed were (top left) amphibians, anthozoans, arachnids, birds, bivalves, and fishes and (top right) hydrozoans, insects, mammals, reptiles, sharks/rays, and snails.

Trade networks of the 12 animal groups were highly variable (figs. S2 to S13) and commonly diverged from the overall patterns (Fig. 1). The trade in some animal groups was also uneven, where the market was dominated by few nations/territories. For example, a large proportion of the trade in CITES-listed wild-caught amphibians comprised exports from Madagascar to the United States, while wild fish were mostly traded between Thailand and Hong Kong. Trends in group-specific trade networks in relation to global wealth/wealth inequality were similarly inconsistent (Fig. 3). However, overall patterns linking greater wealth inequality with higher trade network connectivity (Fig. 1) were evident to some degree in all animal groups, except amphibians (Fig. 3). The response of group-specific trade networks to the global economy was also largely lagged.

**Fig. 4:**
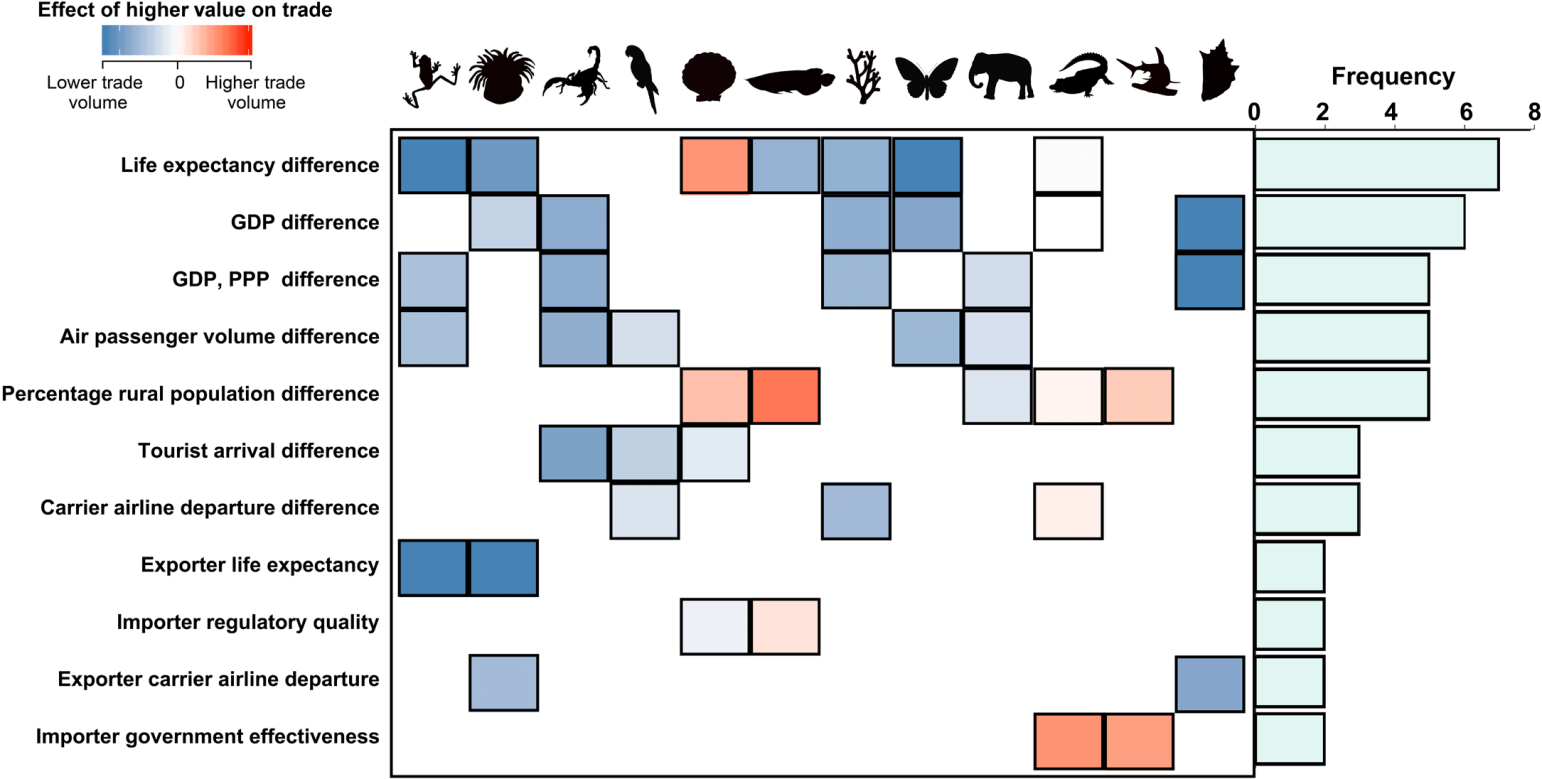
Animal group-specific predictors of bilateral trade volumes. More wild-caught animals were traded between similarly wealthy exporter-importer pairs (i.e., lower socioeconomic disparity) across nearly all groups analyzed. The color of each tile represents slope coefficients indicating the direction and strength of statistical relationships, while the histogram represents the frequency with which respective socioeconomic indicators constitute one of five top variables of importance predicting group-specific bilateral trade volume. Icons represent the animal groups as defined in Fig. 3. This figure has been truncated by removing socioeconomic predictors that were selected as a variable of importance for only a single animal group (full diagram in fig. S14).

At the bilateral level, our group-specific query revealed trends that were consistent with observations from our cumulative dataset (Fig. 1). Specifically, trade volumes in most animal groups were higher between exporter-importer pairs with similar socioeconomic standing (Fig. 4), indicating that comparatively wealthy exporters supplied more wild animal products to the international market. This is likely driven by the same mechanisms underlying patterns in the cumulative trade (see the “Overall trends” section). Other predictors of trade volume were rarely shared among animal groups (fig. S14) as supply-demand dynamics are likely to be unique to each group given differences in source ecosystems, consumer demographics, and logistical requirements (e.g., ease of transportation), among others.

### The bigger picture and study limitations

The data we analyzed in this paper were representative of the legal trade in threatened (i.e., CITES-listed) species, raising questions about the applicability of our findings to the wider international wildlife market inclusive of the illegal trade and trade in nonthreatened (i.e., non–CITES-listed) species. The primary obstacles to this task are the sparsity of quantifiable illegal trade data at the international level (*33*, *35*) and inconsistent record-keeping for non–CITES-regulated transactions. Given these challenges, we contextualized our findings by comparing a subset of the data analyzed (i.e., all trade entering the United States between 2000 and 2014) against the U.S. Fish and Wildlife Services Law Enforcement Management Information System (USFWS-LEMIS)–sourced “WILDb” dataset—the most representative record of trade in the wider international market currently available (*36*). Results from this comparison will provide an approximation of how our findings may apply to the wider global market.

The CITES data we analyzed for this paper represent an estimated 1% of the wider international market. This varied substantially between animal groups, however, and ranged from less than 1% (e.g., for bivalves) to a high of 87% for anthozoans (fig. S15 and table S5). We found that animal groups comprising species traded for food (e.g., fishes and amphibians) were poorly represented by the data analyzed, contrasting with animals traded primarily for hobbyists (e.g., anthozoan and hydrozoan corals; fig. S15). A relatively small proportion of animals were traded illegally across all animal groups (besides sharks and rays), but this is most likely an underestimate because illegal exports/imports are, by their nature, difficult to detect (*35*, *36*).

We also found that trade network properties (i.e., number of trade links) inferred from our data generally scaled positively with the wider international market and the illegal trade (table S5). This broad alignment in trade network patterns across datasets is supported by the fact that illegal trade volumes entering the United States are positively correlated with the wealth of respective source countries (*33*)—reflecting our observations at the bilateral level (Fig. 2). Nevertheless, statistical correlations were weak or reversed (i.e., negative correlation) in some animal groups (table S5). We recommend a conservative reading of our findings, so our observations/inferences should be assumed to be strictly applicable to the legal trade in threatened (CITES-listed) wild animals. Loose generalizations about the illegal trade and the legal trade in non-CITES species could be made for animals belonging to some groups (i.e., denoted by asterisks in table S5), but this should be conditional upon confirmatory studies. Overall, our comparisons further demonstrated the extent of uncertainty in our understanding of the illegal trade and the wider wildlife market.

### Wildlife trade in a post-pandemic world

COVID-19 caused significant global disruptions (*37*, *38*) that are likely to change the landscape of the international wildlife market. For instance, substantial declines in passenger and cargo air traffic (*38*) could be consequential to the wildlife market, as our analyses show that exporter air traffic capacity is positively correlated with bilateral trade volumes. We expect trade volumes to decrease in the short term, especially within the pet market where animals are transported live. While we lack information about the importance of other forms of freight (e.g., ships and trucks) and modes of transportation, we believe that pandemic-related disruptions to these trade routes (*37*) will similarly dampen international wildlife trade because supply chain blockages (*37*) can increase export costs (*23*).

Another pertinent fallout from COVID-19 is the global economic recession that the UNCTAD (United Nations Conference on Trade and Development) estimates to be the deepest since the Great Depression (*37*). Our initial prediction is that this will drive a downward trend in wildlife trade, considering that impacts on global consumption (*38*) may reduce the demand for luxury wildlife products (e.g., exotic pets), while job losses in trade facilitation industries (*37*, *38*) may decrease supply to export markets. However, our findings also show that global trade connectivity could increase if the economic downturn aggravates international wealth inequality by disproportionately affecting certain parts of the world. This, unfortunately, seems a plausible outcome given the severity of socioeconomic impacts in poorer developing nations/territories (*37*).

The pandemic was also a catalyst for regulatory changes, among which China’s ban on wildlife consumption (*39*, *40*) is most notable given its significant role in the global market (Fig. 2). Wildlife trade bans have had drastic impacts on market trends in the past (e.g., the EU’s ban on bird imports), but we do not expect a similarly substantial outcome from the current set of regulations. China’s ban on trade in “terrestrial animals” (*41*) is applicable to 3 (i.e., amphibians, reptiles, and mammals) of the 12 animal groups we analyzed, but its participation in the quantifiable amphibian trade is relatively minor (fig. S2), while the import/export of mammals and reptiles comprises a much smaller proportion of the global trade network than the preregulation involvement of European nations in the bird trade (*29*). On the other hand, China plays a more significant role in the trade of aquatic animals (i.e., fish, hydrozoans, sharks, and rays) (figs. S7, S8, and S12), but these will be unaffected by the new regulations (*41*). At the time of writing, we were not aware of other equally extensive bans, although Vietnam ordered an increase in the enforcement of existing wildlife trade regulations, while the Republic of Korea banned imports of “invasive alien species” (*40*).

Overall, the major post-pandemic changes appear, on balance, to favor a decline in the international wildlife trade. This positive outcome mirrors other adventitious environmental gains from the pandemic (*38*) and should also be viewed as foundations for achieving lasting transformations rather than grounds for returning to business as usual.

### Policy implications

Our findings suggest that socioeconomic drivers of the wildlife market are influential at the global scale, and the absence of socioeconomic incentives/disincentives in current multinational agreements may be limiting our ability to restrict harmful trade practices. On the basis of our observations, we infer that transnational policies aiming to reduce trade in threatened wild animals should (i) account for international socioeconomic inequality to diminish the financial appeal of exporting wildlife products and (ii) increase the export costs for wildlife products.

A socially equitable approach would be to offer source nations/territories financial incentives in return for commitments to reducing wildlife exports. This follows the overarching strategy of the United Nations Framework Convention of Climate Change’s Reducing Emissions from Deforestation and Forest Degradation Program (REDD+), and we propose that funding for the scheme could be similarly raised from wealthier signatory states. This arrangement would provide poorer exporting nations/territories with the resources required to develop alternative sources of livelihood for indigenous communities that depend on the wildlife trade (*20*, *42*).

Incentive-driven policies at the international level also complement local, community-focused frameworks for preventing harmful wildlife trade practices (e.g., poaching) (*43*). The financial incentives can be used to create conditions and support infrastructure that facilitate sustainable uses of wildlife (e.g., ecotourism) (*44*), a potentially costly endeavor. Investments put into developing sustainable livelihoods may also foster a sense of ownership and stewardship, strengthening participation in conservation initiatives (*43*) and/or cooperation in law enforcement activities (e.g., antipoaching patrols) (*44*, *45*). This ensures the longevity of interventions against harmful wildlife trade practices while also protecting the rights of local communities (*43*).

The trickle-down effects of our policy recommendations are important because international wildlife trade agreements may not directly shape domestic markets, which can be more impactful than the international trade in some parts of the world (e.g., the Indonesian songbird market) (*46*). Moreover, some indigenous communities harvest wildlife for sustenance rather than trade (*47*). Advancements in the socioeconomic well-being of these communities could reduce their dependence on wild-caught sources of protein.

We speculate that the global wildlife market is supply constrained (see the “Overall trends” section; Fig. 2), and this means that we expect trade volumes to fall with the successful implementation of supply reduction policies proposed above. Nevertheless, a supply-constrained market can also be characterized by high demand, so we emphasize the need for demand reduction interventions in key importing nations/territories. Demand reduction efforts, which may range from education/outreach to replacing wildlife products with sustainable alternatives, are not only cost-effective (*48*) but they can also engender sustained decreases in the trade (*49*). Supply reduction measures are unlikely to succeed if the demand for wildlife products remains high as persistent demand will cause prices to rise with decreasing supply (*50*), so the potential for greater profits may undermine incentives for leaving the trade.

We caution that a potential limitation of our policy recommendations is the implicit assumption that our goal of reducing trade in threatened species is a desired outcome for all participants of the trade. The wildlife trade can be driven by cultural beliefs (*51*), politics (*52*), or a mix of both. Attempts to change deep-seated cultural practices may be met with resistance (*53*) or be perceived as the imposition of ethical norms by affluent Western societies (*54*). While imperfect, we believe that incentive-driven policies are an upgrade on existing international agreements on the wildlife trade as they may provide source nations/territories with adequate resources for navigating the cultural complexities underlying wildlife harvesting. For example, economic incentives can be used to promote indigenous practices that align with conservation concerns (e.g., hunting taboos), while seeking to minimize those that are potentially harmful (e.g., ritual hunting), with better planning and management (e.g., issuance of limited ritual-hunting permits) (*55*).

The global wildlife trade supports up to “billions” of livelihoods (*56*) and will likely continue in some form for the foreseeable future. Nevertheless, harmful practices within the trade (e.g., unsustainable harvesting and trade in potential zoonotic hosts) are a manifest threat to global biodiversity (*3*) and a pathway for potentially harmful pathogens (*13*), demanding appropriate intervention. The COVID-19 pandemic has brought the wildlife trade back to the forefront of global discourse (*41*). Perhaps this will provide renewed impetus to collective attempts at rectifying the root drivers of the global wildlife market.

## METHODS

### Wild animal trade data

We downloaded all available trade data in the “comparative” format from the CITES database (*57*) comprising annual reports from 1975 to 2018. This amounted to a total of 2,380,629 data points, each describing aggregated trade of the same group, in the same year, between the same exporter-importer pair and the same trade terms (i.e., units denoting the trade). We then filtered for trade records of plant/animals from the wild by selecting data points from sources denoted by the following: (i) W indicating animals/animal parts taken from the wild; (ii) X indicating animals/animal parts taken from marine ecosystems not under the jurisdiction of any state; (iii) R indicating animals taken from the wild as juveniles/eggs, but raised in captivity; and (iv) U or missing entries indicating animals/animal parts of unknown origin. A total of 1,150,713 data points were retained after this step. Lastly, we narrowed our focus further to animals belonging to the following taxonomic classes: (i) Actinopterygii (fish), (ii) Amphibia (amphibians), (iii) Anthozoa (anthozoans), (iv) Arachnida (arachnids), (v) Aves (birds), (vi) Bivalvia (bivalves), (vii) Elasmobranchii (sharks and rays), (viii) Gastropoda (snails), (ix) Hydrozoa (hydrozoans), (x) Insecta (insects), (xi) Mammalia (mammals), and (xii) Reptilia (reptiles). This returned a total of 1,009,695 data points.

We standardized trade volumes by approximating the number of whole animals traded. We did this in four different ways, depending on the unit used for denoting trade volume. In the first category, where trade volume was reported in terms of “number of individuals,” we treated each unit as a single animal. In the second category, where trade volume was reported in terms of body part counts (e.g., skins, skulls, and tusks), we estimated the number of individual animals traded on the basis of the expected number of body parts on a whole individual. For example, one tiger skull represents an individual tiger, while two elephant tusks are the equivalent of an individual elephant. In the third category, where trade volume was represented by weight, we estimated the number of individual animals traded on the basis of the average weight of a whole adult individual. When the data were unavailable, we based our estimation on the weight of close relatives (e.g., congeners and confamilials) or on the lowest reported “maximum weight” with the assumption that individual animals falling within the higher weight percentiles are less common in the trade. For the fourth category comprising trade in animal body parts with trade volume denoted in weight, we estimated the number of body parts involved on the basis of the average weight of an individual part (e.g., a shark’s fin weighs approximately 5% of its overall mass) (*58*) before calculating the number of animals traded using the same procedure as the second group. We based most of our estimates here using wet weight information, given the lack of dry weight data. We excluded data points of trade reported in terms outside of the four groups described, as we were not able to objectively approximate the number of individual animals traded. Examples include reports involving skin fragments, feathers, and scales. Our standardized dataset consisted of 610,305 data points. Data sources for weight-based estimations are listed in table S2 and detailed in data S1.

In “comparative” CITES reports, trade volume may be reported by the importer, the exporter, or both parties. In rare cases where values reported by the importer and the exporter were inconsistent, we took the larger volume as representative of the specific data point to account for possible underreporting by either party. Furthermore, we did not consider reexports in our analyses, as we were interested in the net movement of wild animals in the trade. In entries associated with reexports, the original source of the animals (i.e., under the “Origin” column) was designated as the exporting nation/territory for subsequent analyses.

Last, we aggregated the data by year, importer, and exporter by summing all trade flows regardless of taxonomic identity. This means that each data point described the total number of animals traded between respective exporter-importer pairs in a specific year (i.e., trade volume). We repeated the procedure for animal group-specific data subsets. Here, each data point represented the total number of animals belonging to a respective group that was traded between an exporter-importer pair in a specific year.

### Socioeconomic data

We compiled the following socioeconomic data from the World Trade Organization (WTO) (*59*) and the World Bank (*60*) databases: (i) overall annual merchandise imports, (ii) overall annual merchandise exports, (iii) annual air freight, (iv) annual air passenger volume, (v) annual energy use, (vi) annual GDP in current US$, (vii) annual GDP growth, (viii) annual per capita GDP, (ix) annual per capita GDP growth, (x) gender equality index, (xi) government effectiveness index, (xii) national Gini coefficient (i.e., national income inequality), (xiii) life expectancy, (xiv) political stability index, (xv) poverty index, (xvi) annual GDP converted by a purchasing power parity factor (GDP and PPP), (xvii) primary education completion rate, (xviii) percentage rural population, (xix) regulatory quality index, (xx) rule of law index, (xxi) control of corruption index, (xxii) annual total airline departure, and (xxiii) annual tourist arrival. Information about specific datasets are detailed in table S1. We matched these indices to the importer and exporter nations/states in our animal trade dataset according to year. We maximized data resolution by analyzing territories separately if specific WTO and World Bank data were available (e.g., Hong Kong and Puerto Rico). Otherwise, the territories were grouped under the highest common (i.e., national) jurisdiction (e.g., Guernsey and Falkland Islands under the United Kingdom).

### Statistical analyses

We conducted our analyses at two levels, global and bilateral (i.e., trade between exporter-importer pairs), using data from 1998 to 2018. First, for global analyses, we constructed empirical annual trade networks describing the direction and magnitude of trade flows between participating nations/territories (data S1). We summarized the topology of these networks by calculating the following indices: (i) throughput (i.e., total volume of all trade flows), (ii) connectance (i.e., proportion of possible trade links realized), (iii) link density (i.e., average trade links per participating nation/state), and (iv) total links (i.e., total trade links in the network). We compared this against the following measures of global wealth and wealth inequality: (i) mean GDP, (ii) mean GDP per capita, (iii) global GDP inequality, and (iv) global per capita GDP inequality. We calculated (i) and (ii) by averaging the GDP and per capita GDP values of nations/territories participating in the trade, while (iii) and (iv) were derived from the same sets of values using the ‘gini’ function on the reldist statistical package (*61*). Gini coefficients typically represent income inequality of individuals within a nation/territory, but we apply the concept at a broader global scale to measure inequality in the distribution of GDP and per capita GDP of nations/territories.

Both our network indices and global economic variables comprised time series data (measured from 1998 to 2018), so we controlled for temporal autocorrelation by calculating the differences between consecutive observations (i.e., differencing). We then fit the following linear model to the differenced network indices and global economic variables: *y_t_*~β_0_ + β_1_*x_t_* (Eq. 1). Here, *t* represents the years between 1999 and 2018 (differenced values cannot be calculated for the first year, i.e., 1998), while *y_t_* represents the differenced network indices (e.g., connectance) in the *t*th year and *x_t_* represents differenced measures of global wealth/wealth inequality (e.g., mean GDP) in the *t*th year. We also tested trade network indices against annual measures of wealth/wealth inequality in the preceding year (replace *x_t_* with *x_t−1_* in Eq. 1) to account for potential lag in the response of trade networks to the global economic climate. We scaled all variables before parameterizing the intercepts (β_0_) and slope coefficients (β_1_) using a Bayesian approach. We ran 50,000 iterations of each model (i.e., each combination) with a burn-in of 5000 iterations on four chains (thinning = 10) using the rjags statistical package (*62*). The direction and strength relationships between network indices and measures of wealth/wealth inequality were inferred from the respective slope coefficients.

In the second part of our analyses, which was focused at the bilateral (exporter-importer pair) level, we compared trade volume (i.e., number of individual animals traded) against the socioeconomic indicators of exporters, importers, and the difference between both parties. We started the process by narrowing down the list of predictors from a total of 65 variables (i.e., socioeconomic indicator of exporter, importer, and difference between the pair, with the exception of overall merchandise exports/imports of exporter and importer for the latter, table S1) by selecting the five most informative variables using Random Forest algorithms on the Rborist statistical package (*63*). Next, we fit the following model to our data: log *v_tj_* = β_0_ + β_1_*z_tj_* (Eq. 2), where *v_tj_* represents trade volume associated with the *j*th exporter-importer pair in the *t*th year, while *z_tj_* represents socioeconomic variables of importance for the *j*th exporter-importer pair in the *t*th year. We parameterized the β_0_ and β_1_ coefficients using a negative binomial regression approach for all five top variables of importance with the same settings described for Eq. 1. The direction and strength of relationships between trade volume and socioeconomic predictors were inferred from the respective (exponentiated) slope coefficients. We repeated both parts of our analyses using data subsets for each of the 12 animal groups specified.

### Comparison against the USFWS-LEMIS database

We compared a subset of our data comprising all trade entering the United States between 2000 and 2014 against trade data sourced from USFWS-LEMIS (i.e., the WILDb dataset), covering the same time period. The WILDb dataset was assumed to represent the wider trade market as it includes records of legal trade in nonthreatened (i.e., non–CITES-listed) species, legal trade in threatened species, and illegal trade in threatened species (*36*), although the latter is likely to be an underestimate. From this, we extracted data associated with trade in wild-caught animals (i.e., entries denoted by W or U under the “source” column) from the 12 groups examined to maintain consistency with our analyses. We restricted comparisons to trade records that were quantified in terms of number of individuals or roughly equivalent body part counts (e.g., skulls and full pelts, but not bone and skin fragments) to reduce uncertainty when comparing both datasets.

First, we estimated the proportion of total trade represented in our analyses. Here, we divided our cumulative trade volume data by the corresponding value from the WILDb dataset with the assumption that trade records in the CITES database were accounted for in the more inclusive WILDb dataset. We also compared our trade volume data against the corresponding value in the illegal trade, where the latter was defined as all imports refused by the United States (i.e., denoted by R under the “actions” column in WILDb). Next, we calculated the statistical correlation between the number of trade links in our data and the total number of trade links in the wider international market as well as the illegal trade, respectively. We did this by fitting Bayesian linear models to our data using the rjags statistical package (*62*). We summarized the statistical correlations with slope coefficients indicating the strength and direction of each relationship (i.e., positive or negative). These protocols were repeated across all animal groups analyzed. We conducted data processing, analyses, and visualization on the R statistical environment (version 3.6.2) (*64*).

## SUPPLEMENTARY MATERIALS

Supplementary material for this article is available at http://advances.sciencemag.org/cgi/content/full/7/19/eabf7679/DC1
